# Plastic Filling, and the Basal Principles of the New Departure

**Published:** 1878-04

**Authors:** J. Foster Flagg

**Affiliations:** Philadelphia.


### ARTICLE III.

### *Plastic Filling, and the Basal Principles of the New Departure.*

BY J. FOSTER FLAGG, D. D. S., OF PHILADELPHIA.

Mr. President, and Gentlemen of the Odontological Society of New York.—I wish to thank you, from the very bottom of my heart, for the invitation you have extended to me to speak to you this evening. I wish you to understand that I distinctly appreciate the honor which you have conferred upon me by giving me more than ten times the usual limit given to one who speaks before you. Furthermore, gentlemen, I know that you are well aware that I have much to present to you which is out of the ordinary course of communications to dental societies. But, gentlemen, you don't know half what a heretic I am!

That which I bring you to-night is no growth of a day. It is no work of a year. I therefore recognize that what seems to me to sound as it ought to sound, will sound to you just as it ought not to sound. I shall present to you the time-honored and ordinarily "accepted creed" of dentistry, and I shall advocate before you the diametrically antagonistic "creed of the New Departure."

Do you suppose it is a new thing for me to be antagonizing accepted dentistry? No, gentlemen. For more than twenty years I have not known what it is to be upon the "right side."

Twenty-two years ago, my very good and highly esteemed friend, Prof. Robert Arthur, enunciated his belief in leaving decay in the cavities of teeth and filling over it, and it was stigmatized as nasty, dirty, slouchy work, and our "great man," Prof. J. D. White, said that "when he could not spend time to properly clean out the cavities he would retire from practice." Here is a document, written by my honored father's own hand, giving an account of the action of a college faculty on the question: "When the Faculty of the Old College met for the purpose of arranging the last 'announcement' of that school, exception was taken by Prof. White to what he considered as 'false doctrine' on the part of Prof. Arthur, in regard to two prominent features in our art, both of which may be considered of vital importance to our success as instructors, and to the successful practice of many of our graduates. The first of these was, that Prof. Arthur advocated the leaving of caries in the cavity of a tooth and plugging thereon. And the second, deemed equally objectionable, that of using 'sponge gold' as a material for filling teeth, and as a substitute for gold foil. *Now, although every other member of the faculty fully coincided with Prof. White in his opposition to this practice of Prof. Arthur,*" etc., etc.

How many of the professors of to-day oppose the "leaving of decay and the plugging there on" when the pulp would be exposed by its removal? It is now the accepted practice; and yet, only twenty little years ago, it was so heretical and such "false doctrine" that the entire faculty "coincided with Prof. White," and Dr. Louis Jack and Dr. J. Foster Flagg were the only two men of the forty-five hundred dentists of the United States that immediately accepted Prof. Arthur's practice, and from that day to this have systematically left decay in every cavity where its removal would expose a living pulp. Thus you see, it is no new thing for me to question the creed of the profession. I became used to it when I was young, and it has grown with my growth.

Years have rolled on, and, as you know, without much attendance on my part at dental meetings; but do you suppose I have been doing nothing? Far from it. Day by day I have filled tooth after tooth and have marked what I have done. Year by year I have tabulated results until they now amount to thousands upon thousands. Letters by the score have passed between Syracuse, St. Louis and Philadelphia. At length I wrote to my friends, Drs. Palmer and Chase, that I felt we were as ready now as ever we should be.

Gentlemen, we believe that we have, for our "New Departure," grand, basal, foundation principles, which have so grown with us that they already seem as old and as solid as the hills.

More than twenty years ago, Prof. Elisha Townsend gave to his contemporaries the assertion that he "saw daily the undeniable evidence of the fact that teeth could be saved with amalgam, which *he* could not save with gold." His memory is revered by us all. As a worker in gold he was unsurpassed—as a proof of his estimation of plastic filling he gave to his profession "Townsend's Amalgam," that material with which we began our labors, that material which had so much of good in it that we were more and more impelled, as the years passed by, to recognize its value.

I feel that I owe much to Prof. Townsend, for he made the way of experiment easy for me. Within a very short time after his death, nearly two hundred of his families became my patients. This not only placed me (then a beginner *in Philadelphia*) at once in full practice, but enabled me to cultivate a ground for plastic filling, which had been well broken by one in whom they had unbounded confidence. Now, gentlemen, the statistics which I propose to offer to-night have been based upon this experience.

ARTICLE I. ACCEPTED CREED.—*Gold is the best thing to save teeth.*

ARTICLE I. NEW DEPARTURE.—*In proportion as teeth need saving, gold is the worst material to use.*

Gentlemen, I am, for the moment, literally but a mouthpiece; Dr. Chase has spoken to you. This article is his. He was the first who had the hardihood to say that "in proportion as teeth *need* saving, gold is the *worst* material to use." This he has said, and Dr. S. B. Palmer and I fully endorse that which he has spoken.

Mark my words! This is a real *belief* which I am speaking to you. Men are coming to our belief every day—the thinking men, the observing men of dentistry. They write me that they introduce their beautiful jewels of gold, with the view of their premature wreck staring them in the face! That with their very best and long continued effort, they are unable to save teeth as they would *with gold.*

And is this nothing? Can a man continue to work and put "piece after piece" in proper place when he feels that the work is killing the patient, and himself, too, and that, when a few short years shall have passed away his work will be good for nothing?

This is what sapped me fifteen years ago. I had been filling teeth *then* for fifteen years. I can show you plenty of plugs of gold—not twenty or thirty, but hundreds—that I introduced more than twenty-five years since, and which are just as good to-day as on the day when they were done. But these were in strong teeth, in places easy and accessible, in teeth that might have gone but little had they been left unfilled; while of the others, of the teeth that *needed* saving, I can but tell you just what all have seen. One by one these teeth came back to me. Day by day I saw my best efforts going to destruction. I *heard* that my work must be defective; but I *felt* that there *might be something lacking in the material.*

And then there were the first of my statistics. It is more than twenty years since this work was begun. Twenty-two years ago I made five hundred fillings. Two hundred and fifty were of gold—just as the cavities came in practice,—the next two hundred and fifty were of tin, gutta-percha and amalgam, the amalgam taking the brunt of the battle and used in places that were too far gone for any other thing. I then waited for five years, watching the progress of events, and, meanwhile, returning to orthodox practice. At the end of this time I was not satisfied, and I waited another year. It was to be a *life-work* with me. I could not do it over again, nor can I. I have done!

At the end of that time the "undeniable evidence" presented itself to my mind. I felt the truth of what Dr. Townsend had said, and came solidly to the belief that *I* could *save teeth* with amalgam which I *could not* save with *gold;* for already in the soft teeth, in the inaccessible places, in the cavities under the gum and with frail walls, the gold fillings were becoming defective, while the plastic fillings far, far better held their own.

To-day, gentlemen, the efforts in gold which formed that trial band have almost passed away; and still quite a number of the plastic fillings, *some even of the most hopeless cases*, are pointed to with pride and satisfaction by their owners.

Seventeen years ago I began the systematic introduction of six per cent. of plastic fillings, choosing, of course, those places best adapted for them—that is, *the worst.* The next year I introduced twelve per cent., the next eighteen per cent., and year by year results were such as warranted this progression. You can readily see where this ends. This is my seventeenth year, and I should have put in 102 plastic fillings in every 100. I found myself not equal to the emergency.

I stand before you with a practice which I have been sixteen years in arranging. I have taken the refuse teeth from all directions; announcing, from year to year, to my patients, that I desired the teeth that were ordinarily extracted, and that I was fast abandoning artificial work, because I felt that I could do my patients better service by repairing wrecks and building crowns on roots. The result is, that I have many mouths in which the teeth have proved themselves wretched beyond wretchedness, yet these are all comfortable. I have teeth by the hundreds such as melt (for they *have* melted) from gold like snow before the sun, at the hands of some of our very best operators.

For all this heap of seeming impossibility I use nothing but plastic fillings. Once in a while I use *tin* because I am engaged on a series of experiments to discover the action of this metal on dentine. Our filling materials are gutta-percha, amalgam and oxychloride of zinc (so called.) With these I do everything. All this in the mouths of the *very best* class of patients.

"*In proportion* as teeth *need* saving, gold is the *worst* material to use."

This point alone takes in the whole of Palmer's electro-chemical theory of the action of filling materials: but I must tell you what the work of winnowing out the chaff, as far as we could do it, has eliminated.

The work has been borne by Prof. Henry Morton, of Hoboken, and Prof. Snyder, of Philadelphia, scientists; Messrs. Eckfeldt and Du Bois, Assayers of the Philadelphia Mint, metallurgists, and Drs. Palmer, Chase and Flagg, dentists.

We have carefully, and, we believe, scientifically, investigated every line of experiment which has professed to show *the current* which, we believe, is eliminated by contact of metal with tooth-bone. This we have done with the best instruments and apparatus which our country can produce, —and we find that such current *has never been shown*, much less measured. Even that curiously ingenious line of experimentation upon this subject, which was presented at the "Centennial" meeting of the American Dental Association, and which gave to our profession that scale of relativity known as the "Harvard Tension Series," in which gutta-percha was placed at "0" and gold at "200°," and which gave those who had faith in gold such "a black eye" (as it was expressed,) we pronounce utterly worthless and without any scientific basis. And yet I will read you Prof. Morton's opinion of that electro-chemical theory upon which we rest our belief, as expressed in this Article I; for if gold *is* the worst material to use, there must be some scientific reason.

After speaking of the great difficulty of *current elimination*, Prof. Morton says: "On the other hand, we have the best reason for assuming, on analogy and authority, that all chemical action invokes electrical disturbance. Now acid fluids do act chemically on bone, and the presence of a good conductor, which is itself unattacked, in contact, must, on general electrical principles, assist the electro-chemical action. Supported by your facts of observation, and the reasonableness and consistency of the electric theory, you may well rest your case there. But it is, of course, very important not to give your opponents a chance to attack by resting, for a proof of your theory, on experiments which are themselves unsound and capable of confutation."

Now you see, gentlemen, that notwithstanding the fact that we are unable, as yet, to *demonstrate* the existence of the current, yet, nevertheless, the "reasonableness" and "consistency" of our theory is such, that we may say that gold, being a good conductor and being itself unattacked, when placed in contact with tooth bone must, on general principles, cause excessive electro-chemical action, which action must eventuate in disintegration of tooth tissue; and, therefore, gold is the *worst* material to use when a tooth is of such structure and in such condition as that it needs something which will help to preserve it.

The stereotyped answer to this is couched in these few words: "If gold is so put in as that no fluid can come between it and the tooth bone, there will be no chemical action—because where there is no fluid there cannot be any chemical action." Then, gentlemen, *why don't you put it in so?* If you cannot, then use something else, if for no other reason.

[TO BE CONTINUED.]

